# Evaluation of Habitat Suitability and Assessment of the Invasion Risk of Water Hyacinth [*Eichhornia crassipes* (Mart.) Solms] in Global Freshwater Ecosystems

**DOI:** 10.3390/plants15081279

**Published:** 2026-04-21

**Authors:** Prabhat Adhikari, Pradeep Adhikari, Anil Poudel, Yong Ho Lee, Sun Hee Hong

**Affiliations:** 1 Department of Plant Resources and Landscape Architecture, College of Agriculture and Life Sciences, Hankyong National University, Anseong 17579, Republic of Korea; adprabhat2051@gmail.com (P.A.); aneeelily@gmail.com (A.P.); 2 Institute of Humanities and Ecology Consensus Resilience Lab, Hankyong National University, Anseong 17579, Republic of Korea; pdp2042@gmail.com

**Keywords:** water hyacinth, invasive species, species distribution modeling (SDM), MaxEnt, habitat suitability

## Abstract

Aquatic ecosystems worldwide are increasingly threatened by invasive species, with water hyacinth [*Eichhornia crassipes* (Mart.) Solms] being among the most destructive aquatic weeds. Despite numerous regional studies, a global assessment integrating climatic and hydrological drivers remains lacking. Here, we assessed current and future invasion risks across 55,945 freshwater lakes using the maximum entropy (MaxEnt) model. Climatic variables and key aquatic parameters, including biological oxygen demand (BOD), water depth, and discharge, were incorporated under two shared socioeconomic pathways (SSP2-4.5 and SSP5-8.5). Annual mean temperature, annual precipitation, and BOD were the strongest predictors of habitat suitability. Under current conditions, 5524 lakes, primarily in tropical and subtropical regions, were identified as being suitable habitats, with medium-sized lakes exhibiting the highest proportional suitability (16.54%). Although small lakes were most frequently classified as suitable due to their abundance, larger lakes showed higher suitability intensity. Future projections indicated marked habitat expansion, especially under SSP5-8.5, with suitable lake surface area increasing to 18.12% by 2061–2080. Moreover, 543 currently unsuitable lakes, including Lake Erie, Lake Huron, and Lake Ontario, were projected to face elevated invasion risk, particularly in Africa, South Asia, Southeast Asia, and North America. This global, lake-specific assessment supports early warning, targeted management, and climate-responsive policy planning.

## 1. Introduction

Aquatic ecosystems are highly vulnerable to invasion by nonnative species because of multiple factors, primarily anthropogenic activities such as aquaculture, the aquarium trade, and water-mediated transportation systems [1,2]. Aquatic invasive plants, particularly species such as water hyacinth, significantly disrupt hydrological and ecological processes by increasing evapotranspiration, reducing water availability, and degrading water quality and ecosystem integrity [2,3]. The global economic loss due to aquatic invasions is estimated to reach as high as $345 billion, including assessment and management costs, with aquatic invasive plants alone contributing an estimated $20 billion by 2020 [4].

Water hyacinth [*Eichhornia crassipes* (Mart.) Solms] is a free-floating perennial aquatic plant among the 100 worst aquatic weeds in freshwater ecosystems native to the Amazon Basin in South America [5]. The species was initially intentionally introduced for ornamental purposes and later spread to more than 50 countries in tropical and subtropical regions worldwide [6]. Its highly invasive nature is attributed to its rapid reproduction through both vegetative propagation via stolons and sexual reproduction via seeds, along with its notable ability to double its biomass within six to fourteen days under favorable conditions [7]. Previous studies have revealed that only ten water hyacinth plants can multiply into approximately 655,360 individuals within eight months, potentially covering nearly half a hectare [6,8].

Water hyacinth produces dense floating mats that obstruct sunlight penetration, thereby decreasing photosynthesis in submerged plants, reducing dissolved oxygen levels, increasing turbidity, and causing mortality of native flora [9,10]. Moreover, water hyacinth adversely affects physical infrastructure, such as hydropower systems, by clogging irrigation channels and obstructing water flow [11]. Owing to its fast growth, rapid spread, and resilience in various water bodies, this species is highly challenging to control and manage.

Urban and peri-urban areas function as the primary dispersal hubs for water hyacinth, facilitating its spread into adjacent water bodies. Rapid population growth and urbanization have increased the discharge of nutrient-rich wastewater and agricultural runoff, creating eutrophic conditions that are ideal for water hyacinth proliferation [10,12]. Moreover, global climate change amplifies these threats by increasing temperatures and altering precipitation patterns, thereby expanding the ecological niche that is suitable for water hyacinth invasion [10,13]. Increasing temperatures increase plant growth and survival in previously unsuitable regions, whereas shifting rainfall patterns and extreme weather events facilitate long-distance dispersal and establishment in new habitats [14]. The combination of warmer waters and elevated nutrient loads creates highly favorable conditions for the spread of water hyacinth, posing significant threats to aquatic biodiversity, water security, and socioeconomic livelihoods [12,15].

Therefore, it is essential to determine the habitat suitability of water hyacinth and assess its potential invasion risk under global climate change scenarios and local environmental conditions for early planning of control and management strategies to protect aquatic ecosystems and human livelihoods. More recently, species distribution modeling (SDM) has emerged as a critical approach in freshwater ecology for estimating the spatial and quantitative distributions of aquatic invasive weeds [10,14].

Among the various algorithms employed in SDM, the maximum entropy (MaxEnt) model is among the most widely applied machine learning approaches because of its notable predictive performance and its ability to produce highly accurate and ecologically meaningful results via presence-only species occurrence data and limited sample sizes [16,17]. Moreover, the MaxEnt model is particularly effective for modeling aquatic invasive weeds, as sparse occurrence data can be integrated with climate and hydrological variables, rendering it well suited for scenario-based projections under climate change [18]. MaxEnt has been widely applied in species distribution modeling due to its proven capability to reliably predict potential distribution patterns from presence-only data, making it a valuable tool for assessing the spread of invasive species such as water hyacinth in freshwater ecosystems [19].

Despite the increasing interest in ecology, distribution, and climate-based predictions of water hyacinth, significant research gaps remain, particularly at the global scale. Most existing studies have focused on localized regions or individual countries, thus limiting their ability to reveal broader invasion dynamics [20,21]. Additionally, many studies have focused solely on current distribution patterns without incorporating future climate change scenarios, and important hydrological variables such as the biochemical oxygen demand (BOD), electrical conductivity (EC), and pH, which are known to influence the establishment and spread of water hyacinth [10,14], have been neglected. Therefore, this study aimed to conduct a model-based assessment of the invasion risk of water hyacinth by predicting its global spatial distribution on the basis of both climatic and hydrological data. The specific objectives of this study were (1) to identify the key climatic and hydrological variables influencing the habitat suitability of water hyacinth, (2) to project the current and future global distributions of water hyacinth under climate change scenarios, and (3) to evaluate invasion risk by classifying global lakes into distinct risk categories.

To our knowledge, this study is the first to rely on a modeling approach to evaluate the invasion risk of water hyacinth across 55,945 global lakes under changing climatic conditions. This study is significant because it provides a comprehensive assessment of invasion risk, thereby offering critical insights into early detection, risk mitigation, and the development of effective management strategies to protect freshwater ecosystems worldwide.

## 2. Results

### 2.1. Selection of Modeling Variables and Their Relative Contributions to the Model

Among the 12 variables, Bio1, Bio12, and BOD contributed the most to the model, accounting for 31.10%, 36.94%, and 15.85% of the overall variation, respectively (Table 1). The contribution of altitude was moderate (10.05%), whereas the remaining variables played minor roles. These results indicate that three environmental variables, i.e., the annual mean temperature, annual precipitation, and BOD, are the main variables influencing habitat expansion and increasing the risk of water hyacinth worldwide.

### 2.2. Evaluation of Model Performance and Validation

The prediction accuracy of the MaxEnt (version 3.4.4) model using species occurrence points and input variables was assessed using the area under the curve (AUC), true skill statistic (TSS), and kappa coefficient. The model established with species occurrence data and bioclimatic variables with additional aquatic environmental variables employed as inputs achieved the highest scores across the various metrics, with AUC, TSS, and kappa coefficient values of 0.86, 0.83, 0.70, 0.88, 0.87, and 0.58, respectively, when the test datasets were used (Table 2).

**Figure 1:**
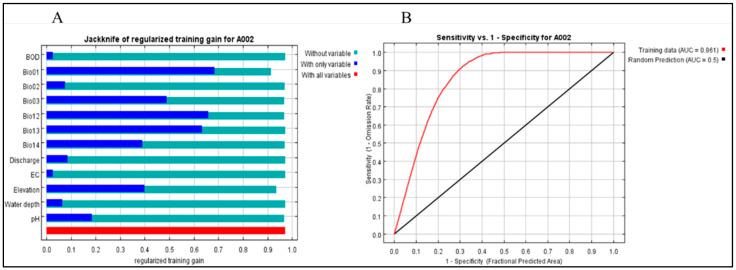
Predicting the relative contributions of bioclimatic and environmental variables in the MaxEnt model using the jackknife test (**A**) and evaluating the model performance using the AUC under the ROC curve (**B**).

The MaxEnt model achieved the best prediction performance for the global spatial distribution of water hyacinth, reflecting excellent model accuracy and a notable match between the observed and predicted outcomes. Similarly, a jackknife test was performed to evaluate the importance and contribution of environmental variables in predicting species distribution, which revealed that three bioclimatic variables, namely, Bio1, Bio12, and Bio13, were relatively highly important in the water hyacinth model (Figure 1).

### 2.3. Classification of Global Freshwater Lakes on the Basis of Their Coverage Area

Habitat presence of global freshwater lakes was predicted for 55,943 freshwater lakes of varying sizes, representing 15.40% of the total global lake surface area under the current climatic conditions. Among them, 15 lakes (e.g., Lake Superior, Lake Victoria, Lake Baikal, and Great Bear Lake) are very large, each with an area greater than 10,000 km^2^ (Table 3). Of these, eight lakes are located in North America, two in Asia, and two in Africa, and the remaining lakes are distributed across the other continents. Similarly, 105 lakes (e.g., Ontario, Erie, and Turkana) are classified as large lakes, 1326 lakes (e.g., Oneida, Toba, and Champlain) as medium, and 54,497 lakes (e.g., Sebago, Torch, and Pyramid) as small, with respective size ranges of 1000–10,000 km^2^, 100–1000 km^2^, and <100 km^2^. The names of these four categories of lakes are listed in Tables S2.1–S2.4. Many lakes remain unnamed and are identified by their assigned ID numbers.

**Figure 2:**
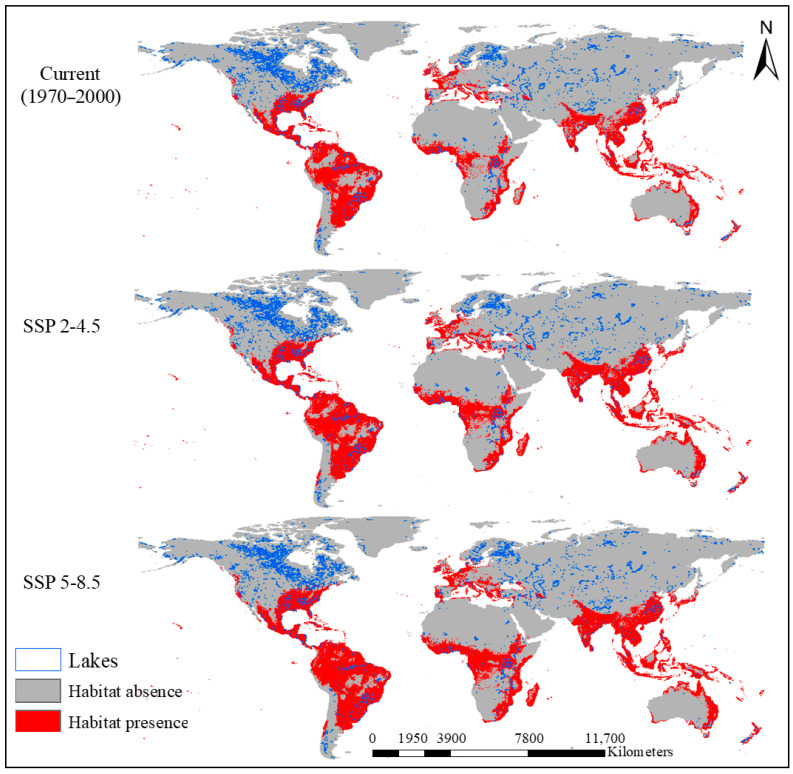
Current and future (2061–2080) habitat suitability levels of Water hyacinth under the SSP2-4.5 and SSP5-8.5 scenarios.

The distribution of water hyacinth is concentrated primarily in lakes in tropical and subtropical regions, with the highest concentrations observed in countries such as Brazil, India, Nigeria, Indonesia, China, and the southern United States (Figure 2). Most invasions occur in small lakes, accounting for 5248 lakes (e.g., Argazinskoye, Kuo-mang, and Zhangdu), largely because of the global abundance of such water bodies (Table 4 and Table S2.1).

**Figure 3:**
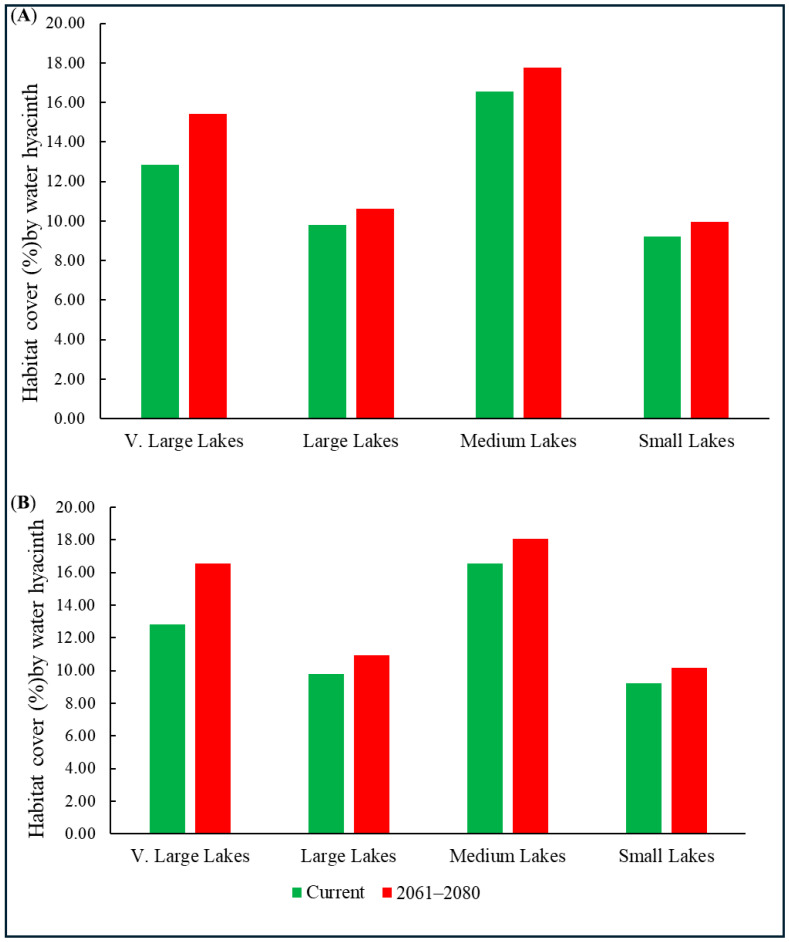
Habitat cover of Water hyacinth across different global lake categories under two climate scenarios: SSP2-4.5 (**A**) and SSP5-8.5 (**B**).

In terms of surface coverage, medium-sized lakes were the most notably impacted, with water hyacinth accounting for 16.54% of their total surface area, followed by very large lakes, at 12.83%, and large lakes, at 9.80% (Figure 3). Among the 15 very large lakes, at least eight (e.g., Lake Tanganyika and Lake Ontario) currently provide climatically suitable habitats (Table 4). Similarly, six of the 105 large lakes (e.g., Lagoa Mirim, Chiquita, and Buenos Aires) were projected to be suitable under current conditions (Table S2.4). This pattern indicates that although small lakes are more frequently invaded, water hyacinth generally occupies a larger proportion of the surface area in medium and very large lakes, potentially exerting greater ecological pressure in these systems.

Overall, 50,433 lakes were predicted to be climatically unsuitable, yet more than 5500 lakes across different continents were classified as providing low to very high suitability for water hyacinth invasion (Table 5). These lakes will become suitable habitats by 2061–2080. The rate of habitat expansion is greater under the SSP5-8.5 scenario than under the SSP2-4.5 scenario. Specifically, under the SSP2-4.5 scenario, suitable habitats are projected to cover 17.35% of the global lake surface area, whereas under the SSP5-8.5 scenario, this value increases to 18.12% from 2061–2080 (Figure 3). Unsuitable, low-, moderate-, high-, and very-high-suitability areas are summarized in Tables S3.1–S3.5.

All the lake categories are projected to experience an increased presence of habitats. Notably, small lakes remained the most frequently affected, with 573 additional lakes projected to become suitable under the SSP5-8.5 scenario (Table 3). By 2061–2080, 20 additional suitable lakes (e.g., Walmsley, Morari, and Pozuelos) are projected to experience gains, while the highest proportional habitat coverage, reaching 18.42% under the SSP5-8.5 scenario, is projected to be maintained. Very large lakes, although fewer in number, exhibited substantial vulnerability, with nine predicted to sustain invasions by 2061–2080 and habitat coverage reaching 17.72%, indicating extensive colonization and potentially greater ecological disruption.

An analysis of habitat suitability revealed that climate change will increase the number of lakes classified as low- to very-high-suitability lakes and decrease the number of unsuitable lakes from 2061–2080. Under the SSP2-4.5 and SSP5-8.5 scenarios, the number of very highly suitable lakes increases sharply, reaching 5780 and 5929 lakes, respectively (Table 4). These findings suggest that ongoing and future climate change will promote the introduction, establishment, and expansion of water hyacinth in numerous lakes worldwide.

### 2.4. Evaluation of the Future Invasion Risk by Classifying Global Freshwater Lakes into Distinct Risk Categories

The global invasion risk of water hyacinth across 55,943 lakes was assessed for the 2061–2080 period under the SSP2-4.5 and SSP5-8.5 climate change scenarios (Table 5). The potential invasion risk was categorized into the following four classes: (1) no invasion risk, (2) stable invasion risk, (3) high invasion risk, and (4) extreme invasion risk. The model results indicated that 49,887 lakes under current climatic conditions, 49,489 lakes under the SSP2-4.5 scenario, and 49,354 lakes under the SSP5-8.5 scenario are projected to remain at the level of no invasion risk from 2061–2080. These lakes are either located within the native range of the species (e.g., the Amazon Basin in South America) or in nonnative regions, such as Lake Baikal (Russia), Lake Superior (North America), and Qinghai Lake (China), but have no documented occurrences of water hyacinth to date (Tables S4.1–S4.4). This category includes lakes ranging in size from small to very large.

In contrast, the model results identified 466 lakes under the SSP5-8.5 scenario as being at an extreme invasion risk. These lakes include Grand Lake, Cayuga Lake, and Fontana Lake in the United States; Lake Joseph and Eagle Lake in Canada; Har Lake in Mongolia; Dianchi Lake in China; and Nerpich’ye Lake in Russia. The highest invasion risk category was concentrated in the Northern Hemisphere, which extended to 60° N latitude. These findings suggest that global climate change is creating new favorable habitats for water hyacinth, thereby posing a threat to the biodiversity of native aquatic flora.

## 3. Discussion

This study aimed to identify the key environmental drivers of water hyacinth distribution and to assess its global invasion risk under current and future climate scenarios. Among the 25 environmental variables considered, highly correlated predictors (r > 0.75) were identified using Pearson’s correlation analysis, and one variable from each correlated pair was removed based on ecological relevance and data quality. Although this approach reduced multicollinearity, some residual correlation among predictors may remain, which could influence the interpretation of variable importance. In this study, occurrence records from both native and invaded ranges were pooled to model the global distribution of water hyacinth. While this approach captures the broad environmental tolerance of the species, it may lead to niche overestimation, particularly if niche shifts or expansions occur in invaded regions. Consequently, the predicted suitable areas should be interpreted as potential distribution rather than confirmed establishment. Future studies distinguishing native and non-native ranges could provide deeper insights into niche dynamics and invasion processes. These findings provide a foundation for understanding how climatic and hydrological processes jointly influence global invasion dynamics.

Our results demonstrate that temperature (Bio1), precipitation (Bio12), and biochemical oxygen demand (BOD) are the primary factors shaping habitat suitability. Under current conditions, approximately 15.40% of the global lake surface area is predicted to provide suitable habitat, with 5381 lakes classified as having high to very high suitability. Under future climate scenarios, particularly SSP5-8.5, habitat suitability is projected to expand poleward, reaching up to 60° N by 2061–2080. This expansion includes 466 newly at-risk lakes, such as Har Lake (Mongolia), Eagle Lake (Canada), and Nerpich’ye Lake (Russia). Notably, even large temperate lakes such as Lake Michigan may become increasingly vulnerable, with approximately 2800 km^2^ projected to become suitable. These findings suggest that climate change is likely to reduce thermal constraints and facilitate the spread of water hyacinth into previously unsuitable high-latitude freshwater ecosystems.

Although numerous studies have examined water hyacinth invasion in the context of climate change, most have focused on individual lakes (e.g., the Nyanza Gulf of Lake Victoria and Lake Tana [22], or have provided global predictions based solely on current climatic conditions without incorporating key aquatic variables such as BOD [10,12,14]. To address this limitation, our study integrates both current and future climate scenarios (SSP2-4.5 and SSP5-8.5) with aquatic environmental variables, including BOD, water pH, and electrical conductivity (EC), to provide a more comprehensive global assessment of habitat suitability and potential expansion across freshwater ecosystems.

Among the bioclimatic variables, Bio1 and Bio12 were identified as key factors influencing the distribution of water hyacinth. Temperature (Bio1) plays a critical role in shaping its survival and expansion, with optimal growth occurring between 25 °C and 30 °C, where metabolic activity, photosynthesis, and nutrient uptake are maximized [23,24]. Higher temperatures further enhance nutrient uptake, particularly nitrogen and phosphorus assimilation, which supports faster vegetative growth and increased chlorophyll production, thereby enhancing its invasive potential [23]. In contrast, growth slows significantly at lower temperatures (<10 °C) due to reduced metabolic activity, limiting survival [23], whereas temperatures above 30 °C may induce thermal stress, although the species remains adaptable up to 40 °C [25]. Our response curves confirm this optimal thermal range, consistent with previous studies. As temperatures rise under future climate scenarios, thermal constraints in temperate regions are expected to decline, thereby reinforcing the projected expansion into previously unsuitable regions through competition with native species and alterations in freshwater ecosystem structure [26,27].

Our study highlights that Bio12 plays a crucial role in shaping the distribution and spread of water hyacinth in freshwater ecosystems. Areas with higher and more variable rainfall support greater abundance, indicating that precipitation is a key driver of invasion dynamics. This pattern is consistent with studies showing that seasonal rainfall is strongly correlated with biomass peaks, particularly in regions receiving more than 1500 mm of annual precipitation [28]. Increased rainfall enhances nutrient runoff from agricultural and urban landscapes, creating eutrophic conditions that promote rapid plant growth [29]. These nutrient-rich environments enable the formation of dense floating mats that outcompete native aquatic plants by reducing light availability and disrupting aquatic food webs [30]. In addition, extreme precipitation events increase hydrological connectivity, facilitating dispersal, while droughts promote fragmentation and subsequent recolonization when water levels rise [29]. This tolerance to fluctuating hydrological conditions, combined with rapid vegetative reproduction, enhances the species’ invasion potential [10,31]. Collectively, these findings indicate that precipitation influences both physiological performance and spatial expansion, contributing to the ecological dominance of water hyacinth.

In addition to temperature and precipitation, other environmental factors influence the establishment of water hyacinth. The species can survive at diverse water depths (0.5–3 m), enabling it to spread across different aquatic environments [32,33], thus emphasizing that water hyacinth is tolerant to a broad range of pH values (5.0–9.0), allowing it to colonize various freshwater systems. This species can persist in both nutrient-rich and nutrient-poor water, reducing the influences of BOD and EC on its distribution [34]. Nutrient pollution, particularly from agricultural runoff, increases water hyacinth invasion by increasing biomass production [35]. Additionally, elevation indirectly affects the distribution by influencing local temperature and precipitation patterns, further supporting the dominant role of climate in determining habitat suitability [36]. Climate-induced shifts in elevation gradients may facilitate the invasion of water hyacinth into new territories.

Our study revealed that under the current climatic conditions, approximately 5524 lakes contain suitable habitat for water hyacinth. These lakes primarily occur in tropical and subtropical regions and cover 15.4% of the global lake surface area. However, future projections under the SSP5-8.5 scenario indicated substantial expansion, with invasions potentially increasing to 18.12% of the global lake coverage by 2100. This expansion conforms to the growing body of literature suggesting that increasing global temperatures, reduced winter mortality, longer growing seasons, and elevated CO_2_ levels will enable many invasive species to expand their ranges poleward [37,38]. Furthermore, increasing temperatures and shifting precipitation patterns are projected to accelerate biological invasions, and these patterns have been shown to increase the range of invasive species [39]. Additionally, increasing temperature extremes may enhance the metabolic activity of water hyacinth, thereby further supporting its projected range expansion [40,41].

Hydrological connectivity plays a critical role in shaping the global spread of water hyacinth by linking otherwise isolated freshwater systems. Flood pulses, river networks, and canal systems facilitate long-distance dispersal of plant fragments, enabling rapid expansion across regions such as the Yangtze River Basin in China, the Nile River system in East Africa, and monsoon-influenced waterways in India and Southeast Asia [23,42,43,44,45]. As a result, water hyacinth can colonize distant ecosystems, including areas where climatic conditions alone may not fully support its persistence. Consequently, once a single lake or wetland is invaded, adjacent water bodies become increasingly vulnerable, highlighting the need for integrated watershed-level monitoring and management.

In addition to hydrological connectivity, lake morphology and nutrient availability further influence invasion patterns. Small and medium-sized lakes are more frequently invaded due to their shallow depth, high abundance, and proximity to nutrient sources such as agricultural and urban runoff [46,47]. However, our analysis (Table 4 and Table 5) indicates that medium and very large lakes often exhibit greater surface area coverage once invaded, suggesting more intense local impacts. In large lakes, invasion severity is further amplified by high precipitation and nutrient loading, particularly in regions such as the Amazon, Congo, and Mekong basins [10,48].

Geographically, climate models have predicted clear northward and poleward shifts in habitat suitability, with future expansion greatly affecting temperate regions that were previously unsuitable for water hyacinth. This trend is shown in Figure 2, which reveals increased red areas in North America (northern United States and southern Canada), Europe (e.g., France, Germany, and Italy), and northern Asia (e.g., China, Russia, and Kazakhstan). Countries projected to experience the greatest future increases include Brazil, India, the United States, China, and Indonesia because of their combination of existing occurrences and expanding suitable habitats. Specific large and high-risk lakes that could be significantly affected include Lake Victoria (East Africa), Lake Taihu (China), Lake Maracaibo (Venezuela), Lake Okeechobee (USA), and Tonle Sap (Cambodia), although further geospatial analysis is needed for precise identification.

Similarly, larger lakes such as Lake Victoria (East Africa), Lake Tana (Ethiopia), and Lake Chapala (Mexico) were historically less invaded because of their size and deeper water columns, which reduce nutrient concentrations. However, these lakes are now increasingly at risk because of their long nutrient retention times, elevated CO_2_-driven productivity, and human-mediated dispersal via boat traffic and river networks [49]. For instance, Lake Victoria has experienced repeated hyacinth outbreaks because of the high nutrient loading stemming from surrounding urban and agricultural zones and favorable hydrological pathways [49,50].

Our risk assessment results (Table 4) revealed that the invasion potential of water hyacinth is increasing globally, especially under future climate scenarios such as SSP5-8.5. The number of lakes with very high suitability for invasion is projected to increase from 5317 at present to 5929 by 2061–2080, whereas the number of unsuitable lakes is projected to decrease. Our results suggest that increasing temperatures, fewer frost days, longer growing seasons, and increased precipitation drive this trend, which promotes water hyacinth survival and expansion [51]. Although water hyacinth is often used in water pollution control projects because of its ability to absorb nitrogen and phosphorus, it also poses serious risks if not properly managed. Water hyacinth can remove more than 80% of nitrogen and phosphorus from polluted water and is often used to treat wastewater, sewage, and leachate. The plant also removes heavy metals such as copper and lead. However, water hyacinth can store large amounts of these pollutants in its tissues. When it dies or decomposes, those pollutants can return to the water or soil, thereby causing secondary pollution [52]. This process can worsen water quality instead of improving it, especially if the plant is not removed regularly.

The effects on soil and sediment are also harmful. When water hyacinth forms large mats, it blocks sunlight and reduces the oxygen content of water. This leads to poor conditions for fish, insects, and native plants. The thick layer of dead plant material on the lake bottom also alters the chemical composition of the sediment, increasing the levels of phosphorus and ammonia [53]. These changes can continue to cause water pollution long after the plant is removed. In both regional and global contexts, nutrient pollution remains a key driver, with agricultural hot spots observed. The economic and ecological risks include infrastructure damage, biodiversity loss, and disruptions to fisheries and hydropower systems, highlighting the urgent need for proactive management strategies such as satellite monitoring, biological control, and stricter nutrient regulations [54]. Owing to climate change, nutrient pollution, and water movement, the spread of water hyacinth has continuously increased in different countries worldwide [55,56]. The increasing temperatures under the SSP5-8.5 scenario reduce winter die-off in northern lakes, allowing water hyacinth to establish in new areas [55]. The surface temperature in Lake Erie has continuously increased by 1.5 °C since 1995, which has extended the warm season and promoted the spread of water hyacinth [57].

To manage these threats, new detection and control strategies are being explored. Advanced satellite monitoring, such as NASA’s Earth Surface Mineral Dust Source Investigation (EMIT) satellite, has demonstrated 92% accuracy in identifying hyacinth mats in Lake Erie [58]. Precision agriculture techniques, such as global positioning system (GPS)-guided fertilizer application, have been demonstrated to reduce phosphorus runoff by 30%, thereby decreasing nutrient pollution in affected watersheds [59]. Additionally, biological control methods, such as the introduction of fungal pathogens such as *Cercospora piaropi*, have successfully reduced hyacinth biomass levels by 40% in Florida trials and may offer a viable solution for northern lakes [60].

The invasion of water hyacinth poses severe ecological and economic threats, including water quality decline, oxygen depletion, and biodiversity loss [61]. It outcompetes native vegetation, disrupts aquatic food webs, and contributes to the establishment of anoxic conditions, endangering fish and invertebrates [62]. The economic burden on fisheries and tourism continues to increase [63]. Moreover, water hyacinth affects crop production by invading agricultural land adjacent to lakes and adversely influences livestock by covering grazing land [64].

Addressing these challenges requires early detection and improved management strategies. Satellite monitoring and artificial intelligence (AI)-based prediction models can help track plant spread and enable rapid responses [65]. Controlling nutrient pollution, particularly through agricultural runoff management, can slow the expansion of water hyacinth by limiting its primary growth drivers [66]. Integrated pest management (IPM), in which mechanical removal, herbicide application, and biological control are combined, offers a more cost-effective and sustainable long-term solution [67].

If left unmanaged, water hyacinth will continue to spread, thereby increasing control costs, degrading freshwater ecosystems, and causing significant economic losses. Effective policies, investment in advanced monitoring technologies, and the promotion of sustainable management practices are essential for mitigating its impact. Proactive intervention is crucial for preserving aquatic ecosystems, maintaining economic stability, and preventing further environmental damage.

Despite several findings, some limitations should be acknowledged. The MaxEnt model relies on presence-only data, which may introduce sampling bias and does not account for true absence information. In addition, the assumption of niche equilibrium (or conservatism) may not fully capture the ongoing spread and potential adaptive responses of water hyacinth in newly invaded regions. Although efforts were made to reduce spatial bias, uneven sampling effort in occurrence data may still influence model predictions. Moreover, important ecological processes—including dispersal limitation, propagule pressure, and biotic interactions such as competition, herbivory, and human management—were not explicitly incorporated, despite their significant role in species establishment and spread. Occurrence records from both native and invaded ranges were pooled to represent the global environmental niche; while this approach captures the broad environmental tolerance of the species, it may lead to niche overestimation if niche shifts or expansions occur in invaded regions. Therefore, the predicted suitable areas should be interpreted as potential rather than realized invasion risk. Future research should aim to distinguish between native and invaded ranges and integrate additional ecological processes, long-term monitoring data, and coupled modeling approaches to improve prediction accuracy and ecological interpretation.

This study provides a novel global-scale assessment of the environmental drivers and invasion risk of water hyacinth using a MaxEnt modeling framework that integrates multisource occurrence data with bioclimatic and aquatic environmental predictors. By extending beyond previous regional analyses, the results offer new insights into how temperature, hydrological connectivity, and nutrient-rich conditions shape global habitat suitability. Despite these limitations, the integration of climatic and aquatic variables enhances the ecological realism of the model and provides a robust global baseline for identifying regions vulnerable to invasion. Future climate projections indicate a substantial increase in invasion risk, particularly in temperate regions where warming, eutrophication, and hydrological alterations are intensifying. Overall, these findings improve our understanding of the global invasion dynamics of water hyacinth and support the development of early detection, monitoring, and proactive management strategies aimed at mitigating its ecological and socioeconomic impacts under changing environmental conditions. These insights are particularly relevant for policymakers and conservation practitioners aiming to prioritize surveillance and control efforts in emerging high-risk regions.

## 4. Materials and Methods

### 4.1. Data Preparation

**Figure 4:**
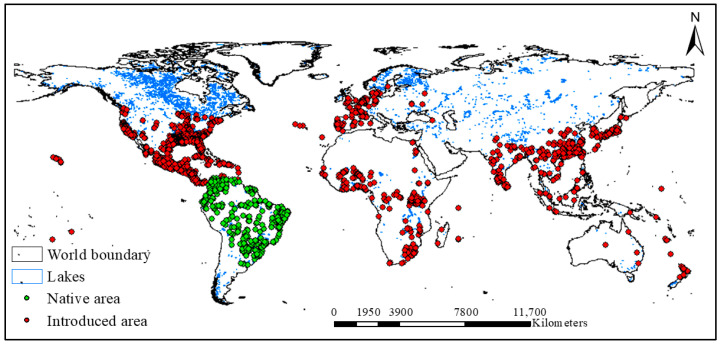
Global occurrence points of water hyacinth obtained from the Global Biodiversity Information Facility (GBIF). The red dots indicate species occurrence points in the introduced area, and the blue areas indicate the boundaries of global lakes.

A total of 17,529 occurrence records of water hyacinth were obtained from the open-source data portal of the Global Biodiversity Information Facility (GBIF) [68]. Occurrence data from both native and invaded ranges were combined to capture the full environmental niche of the species at a global scale. The GBIF provides species occurrence data derived from nonsystematic sources such as museum specimens, administrative records, and citizen science contributions [69]. However, these sources may contain various errors. To minimize spatial bias, remove duplicates, and prevent model overfitting, we manually assessed the records and removed those with improper data sources, missing reporting dates, or locations in dryland areas. We then applied the spatially rarefy occurrence tool in SDM Toolbox v.2.4 within ArcGIS (version 10.8), using a 2.5-min grid cell resolution, as in our previous studies [70,71]. This spatial rarefaction approach reduces spatial clustering and sampling bias, thereby minimizing the effects of spatial autocorrelation in the occurrence data. The implementation of this rarefaction process reduced the total number of occurrence points to 2627, and only one record was retained per grid cell at the specified resolution [72]. Lake boundaries were obtained from the Global Lakes and Wetlands Database (GLWD) [73]. The occurrence points and world boundaries are shown in Figure 4.

### 4.2. Environmental Variables

Climatic and other environmental variables play important roles in driving the invasion of alien weeds. Temperature and precipitation notably influence the establishment of alien species. Therefore, data for 19 bioclimatic variables and the elevation at a spatial resolution of 2.5 min (approximately 4.5 km at the equator) were downloaded from historical climate data for 1979 and 2013. For the future climate change scenarios, we obtained the Max Planck Institute for Meteorology (MPI-ESM1-2-HR) global circulation model (GCM) [74], which was downloaded from WorldClim [75]. This GCM is a high-resolution global climate model developed by the Max Planck Institute for Meteorology for Phase 6 of the Coupled Model Intercomparison Project (CMIP6). Herein, atmosphere, ocean, land, and biosphere components are coupled to simulate climate processes with increased spatial detail. The model has demonstrated notable performance in simulating historical climate and projecting future climate under various emission scenarios [76]. Current climate data and future climate data under the shared socioeconomic pathway (SSP) 2-4.5 and 5-8.5 scenarios for the 2041–2060, 2061–2080, and 2081–2100 periods were used.

In addition to climate data, six environmental variables, namely, BOD and EC, were obtained from NASA Earth data [77]; water depth, water discharge, and pH data were sourced from the Global Environment Monitoring System [78]; and altitude data were acquired from WorldClim [75]. To prevent multicollinearity and improve model performance, Pearson’s correlation coefficients (r ≤ 0.75) were calculated, and three precipitation-related variables (Bio12, Bio13, and Bio14), three temperature-related variables (Bio1, Bio2, and Bio3), and six additional environmental variables (BOD, water depth, water discharge, EC, water pH, and altitude) were selected for modeling the spread of water hyacinth [79] (Table S1).

### 4.3. Model Development

The global spatial distribution of water hyacinth under current and future climate scenarios was predicted using MaxEnt version 3.4.4 [80], based on 2627 occurrence records and background points. Background points were generated using ArcGIS 10.8 (ESRI, Redlands, CA, USA) following established methodologies [81,82]. To evaluate model performance, occurrence data were randomly split into training (75%) and testing (25%) subsets, as recommended in previous studies [83,84].

The modeling process was replicated 100 times to reduce the influence of random data partitioning and improve the robustness of predictions. We applied the default parameter settings in MaxEnt, including a regularization multiplier of 1 and automatic feature selection. Although parameter tuning can enhance model performance, default settings were selected due to the global scale of the analysis and the large number of predictor variables, which increase computational demands and may reduce model transferability. Previous studies have also demonstrated that default settings provide reliable and stable predictions across broad spatial extents [85,86].

### 4.4. Model Evaluation and Validation

Model performance was evaluated primarily using the AUC of the receiver operating characteristic (ROC) curve, which is a widely adopted threshold-independent metric for assessing the discriminatory ability of species distribution models. The AUC reflects the ability of the model to distinguish between presence and background locations, with values ranging from 0.5 to 1.0 [87]. AUC values were categorized as follows: fail (0.5–0.6), poor (0.6–0.7), fair (0.7–0.8), good (0.8–0.9), and excellent (0.9–1.0) [88]. Importantly, the AUC is not affected by the size of the dataset, which makes it a convenient tool for comparing models across studies with varying sample sizes. However, one limitation is that equal weights are assigned to omission errors (failing to predict actual occurrences) and commission errors (predicting the presence where the species is absent), which may not always reflect the ecological or management priorities of a given study [89]. Moreover, the AUC may not fully capture the spatial accuracy of predictions and can provide overly optimistic assessments, particularly when presence data are spatially biased or when pseudoabsence/background points are not carefully selected [89,90]. Therefore, we applied the TSS and Cohen’s kappa coefficient [90] to evaluate the model performance.

The TSS serves as a comprehensive metric for model evaluation, effectively integrating both sensitivity and specificity into a single index, with possible values ranging from −1 to +1 [90]. This metric is particularly valued for its balanced consideration of omission and commission errors, which enables a nuanced assessment of prediction performance. Similarly, the kappa coefficient reflects the agreement between the observed and predicted classifications, with values ranging from −1 (indicating complete disagreement) to +1 (denoting perfect agreement), thereby reflecting model reliability beyond random chance [91]. Superior values for both the TSS and kappa coefficient indicate enhanced model accuracy and robustness, highlighting their importance as core validation measures in ecological modeling.

To better understand the contributions of additional aquatic environmental variables such as BOD, EC, water depth, water discharge, pH, and elevation, we developed two sets of models. The first set was developed using only bioclimatic variables, whereas the second set incorporated both bioclimatic and environmental variables. The average prediction performance of these models was evaluated via three model evaluation metrics, namely, AUC, TSS, and kappa. We then compared the prediction performance between the two model sets to assess whether the inclusion of the six environmental variables increased model accuracy and provided additional ecological insights beyond those explained by bioclimatic variables alone. The model set with the highest prediction performance was used to predict the global distribution of water hyacinth.

### 4.5. Classification of Global Freshwater Lakes and Prediction of Habitat Suitability for Water Hyacinth in Different Categories of Lakes

Studying the invasion risk of water hyacinth in freshwater lakes is critical because these lakes serve as major reservoirs of biodiversity, water supply, and ecosystem services, and their closed hydrological nature renders them highly vulnerable to biological invasions. Unlike flowing or periodically flooded wetlands, freshwater lakes often exhibit limited water exchange, allowing invasive species such as water hyacinth to establish persistent populations once introduced. Therefore, we studied the habitat suitability and invasion risk of water hyacinth in global freshwater lakes. Here, we classified global freshwater lakes by their surface area to better understand their habitat suitability and evaluate the potential distribution of water hyacinth across diverse aquatic environments. Lakes of different sizes exhibit distinct ecological characteristics, including variations in water temperature, nutrient dynamics, and hydrological connectivity, all of which critically influence the spread, establishment, and long-term persistence of invasive species [92]. In this study, a total of 55,945 lakes worldwide were categorized into four size classes, namely, very large lakes (>10,000 km^2^), large lakes (1000–10,000 km^2^), medium lakes (100–1000 km^2^), and small lakes (<100 km^2^), on the basis of their surface area (Table 6). This standardized classification provides a robust framework for assessing habitat suitability, facilitating large-scale ecological comparisons, and predicting the future distribution of water hyacinth under changing climate scenarios.

Probability distribution maps were obtained from the MaxEnt model under the current and future climate change scenarios and were applied to predict the global spatial distribution of water hyacinth. A binary distribution map representing the presence or absence of water hyacinth was created using the threshold maximum training sensitivity and specificity, Cloglog [93]. The resulting global binary distribution maps represent the lakes that are identified as either suitable or unsuitable for water hyacinth within different categories (e.g., very large, large, medium and small lakes), under the current and future climate change scenarios. Afterward, the number of suitable habitat cells was estimated in each lake using zonal statistics tools in ArcGIS Desktop 10.8 (Esri, Redlands, CA, USA), and the proportions of suitable habitat coverage were estimated and classified into five categories, namely, unsuitable, marginally suitable, moderately suitable, highly suitable, and extremely highly suitable, on the basis of average habitat suitability values of 0, <0.25, 0.25–0.50, 0.50–0.75, and >0.75, respectively.

### 4.6. Estimation of the Invasion Risk of Water Hyacinth Under Global Climate Change

**Figure 5:**
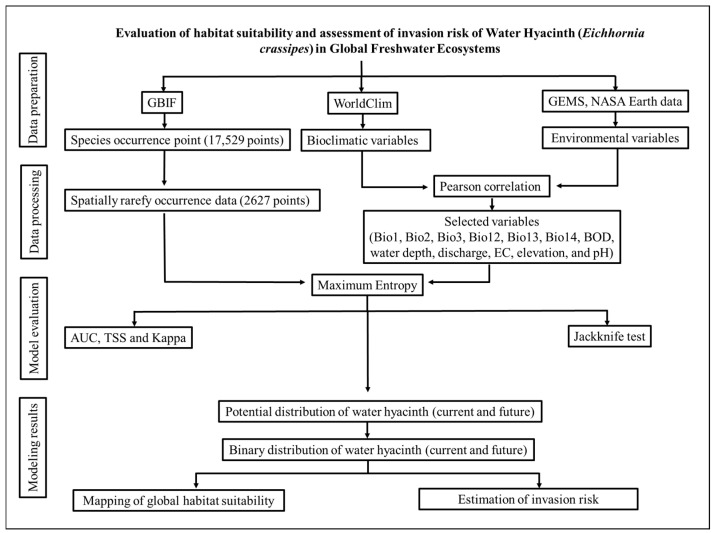
Flowchart of the overall research process, including database management, species distribution modeling, and mapping of water hyacinth habitat suitability.

The potential invasion risk of water hyacinth was classified into no invasion risk, stable invasion risk, high invasion risk, and extreme invasion risk on the basis of the change in habitat suitability under global climate change. Countries within the native range of water hyacinth (South America) and in areas predicted to be climatically unsuitable were classified as having no invasion risk. Lakes lacking current occurrence records but predicted to be climatically suitable for water hyacinth invasion under the current climatic conditions were assigned to the potential invasion risk category. Lakes where the average habitat suitability remained consistent between 1979–2013 and 2061–2080 were designated as having a stable invasion risk. The high invasion risk category refers to situations where the lake status shifts from unsuitable to suitable habitats at a rate of up to 50%. The extreme invasion risk category indicates that lakes are experiencing a transition from unsuitable to suitable habitats, exceeding 50% (Figure 5).

## 5. Conclusions

This study provides the first global, lake-level assessment of the habitat suitability and invasion risk of water hyacinth *Eichhornia crassipes* by integrating bioclimatic, topographic, and aquatic environmental variables under current and future climate scenarios. Our results reveal that the annual mean temperature, annual precipitation, and BOD are the main predictors of the global distribution of water hyacinth. Under the current climatic conditions, approximately 15.4% of the global lake surface area and more than 5500 lakes provide suitable habitats, with occurrences concentrated in tropical and subtropical regions. Future projections indicate a substantial expansion of suitable habitats, particularly under the SSP5-8.5 scenario, with the proportion of suitable areas increasing to more than 18% of the global lake area by 2061–2080. Habitat suitability is predicted to shift poleward, reaching up to 60° N latitude and exposing many temperate lakes that were previously considered safe. The largest proportional increases in surface coverage are observed for medium and very large lakes, whereas thousands of small lakes remain highly vulnerable because of their global abundance and proximity to nutrient-rich landscapes. Overall, 466 lakes, including major systems in Mongolia, Canada, China, and Russia, are projected to face extreme invasion risk by the end of the century. These findings highlight that climate change, combined with hydrological connectivity, nutrient loading, and lake morphology, will significantly accelerate the worldwide spread of water hyacinth. Large, interconnected, and heavily eutrophic lakes are likely to experience the highest levels of ecological pressure. Therefore, early detection, integrated watershed-level management, and effective international cooperation are essential for reducing future invasions and protecting freshwater biodiversity in a rapidly warming world.

## Figures and Tables

**Table 1:** Contribution of environmental variables in species distribution modeling of water hyacinth.

Code	Description	Unit	Model Contribution (%)	Permutation Importance (%)
Bio1	Annual mean temperature	°C	31.10	69.66
Bio2	Mean diurnal temperature range	°C	0.50	0.25
Bio3	Isothermality (BIO2/BIO7) (×100)	%	2.70	2.68
Bio12	Annual precipitation	mm	36.94	7.81
Bio13	Precipitation in the wettest month	mm	0.49	3.95
Bio14	Precipitation in the driest month	mm	0.94	1.98
BOD	Biological oxygen demand	mg/L	15.85	2.03
Depth	Water depth	m	0	0.01
Discharge	Water discharge	m^3^/S	0.88	0.04
EC	Electrical conductivity	S/m	0.01	0.03
pH	Water pH	–	3.54	3.40
Altitude	Altitude	m	10.05	8.15

**Table 2:** Prediction performance of the MaxEnt model for water hyacinth distribution simulation with and without the use of aquatic environmental variables.

Evaluation Parameter	Using Bioclimatic Variables Only	Using Bioclimatic and Additional Aquatic Environmental Variables
AUC	0.752	0.86
TSS	0.68	0.70
Kappa	0.41	0.58

**Table 3:** Estimating habitat presence in different categories of freshwater lakes under the global climate change scenario.

Lake Type	No. of Lakes	Current	SSP2-4.5	SSP5-8.5
Very large	15	6	6	7
Large lakes	105	6	6	6
Medium lakes	1326	262	275	282
Small lakes	54,497	5248	5683	5821
Total	55,943	5522	5970	6116

**Table 4:** Number of lakes based on habitat suitability under current and future climate scenarios from 2061–2080.

Habitat Suitability ^a^	Current	SSP2-4.5	SSP5-8.5
Unsuitable	50,433	49,987	49,839
Low	46	31	40
Moderate	83	89	84
High	64	56	50
Very high	5329	5780	5929

^a^ The different categories of habitat suitability are detailed in Table S2.

**Table 5:** Estimating the number of lakes categorized into different levels of invasion risk under global climate change scenarios.

Invasion Risk Category ^a^	SSP2-4.5	SSP5-8.5
No invasion risk	49,489	49,354
Stable invasion risk	3875	3872
High invasion risk	57	77
Extreme invasion risk	352	466

^a^ Different categories of invasion risk. No invasion risk indicates the number of lakes with no occurrence of water hyacinth and no suitable habitats in the future. Stable invasion risk indicates lakes where the average habitat suitability remains consistent between 1979–2013 and 2061–2080. The high invasion risk category refers to situations in which the lake status shifts from unsuitable to suitable habitats at a rate of up to 50%. The extreme invasion risk category indicates that lakes are experiencing a transition from unsuitable to suitable habitats, exceeding 50%.

**Table 6:** Classification of global freshwater lakes on the basis of their total coverage area.

Lake Type	Area (km^2^)	No. of Lakes
Small Lakes	<100	54,497
Medium Lakes	100–1000	1326
Large Lakes	1000–10,000	105
Very Large Lakes	>10,000	17

## Data Availability

The original contributions presented in this study are included in the article/Supplementary Materials. Further inquiries can be directed to the corresponding authors.

## Supplementary Materials

The following supporting information can be downloaded at: https://www.mdpi.com/article/10.3390/plants15081279/s1, Table S1: Pearson’s correlation analysis of the environmental variables. Table S2.1. List of small lakes (<100 km^2^) worldwide. Table S2.2. List of medium-elevation lakes (100–1000 km^2^) worldwide. Table S2.3. List of medium-sized lakes (1000–10,000 km^2^) worldwide. Table S2.4. List of very large lakes (>10,000 km^2^) worldwide. Table S3.1. List of unsuitable lakes for water hyacinth under the current and future climate change scenarios from 2061–2080 worldwide. Table S3.2. List of low-suitability lakes for water hyacinth under the current and future climate change scenarios from 2061–2080 worldwide. Table S3.3. List of moderately suitable lakes for water hyacinth under the current and future climate change scenarios from 2061–2080 worldwide. Table S3.4. List of highly suitable lakes for water hyacinth under the current and future climate change scenarios from 2061–2080 worldwide. Table S3.5. List of very highly suitable lakes for water hyacinth under the current and future climate change scenarios from 2061–2080 worldwide. Table S4.1. List of freshwater lakes with no risk under the current climatic conditions and future climate change scenarios SSP2-4.5 and SSP5-8.5 by 2061–2080. Table S4.2. List of lakes with a stable invasion risk under global climate change and environmental conditions. Table S4.3. List of lakes with a high invasion risk under global climate change and environmental conditions. Table S4.4. List of lakes with a high invasion risk under global climate change and environmental conditions.
